# Soluble ectodomain CD163 and extracellular vesicle-associated CD163 are two differently regulated forms of ‘soluble CD163’ in plasma

**DOI:** 10.1038/srep40286

**Published:** 2017-01-13

**Authors:** Anders Etzerodt, Ronan M. G. Berg, Ronni R. Plovsing, Morten N. Andersen, Magali Bebien, Mohamed Habbeddine, Toby Lawrence, Holger J. Møller, Søren K. Moestrup

**Affiliations:** 1 Department of Biomedicine, University of Aarhus, Aarhus, Denmark; 2 Centre d’Immunologie de Marseille-Luminy, Aix Marseille Université UM2, Inserm, U1104, CNRS UMR7280, Marseille, France; 3 Centre of Inflammation and Metabolism, Rigshospitalet, Copenhagen, Denmark; 4 Department of Intensive Care, Rigshospitalet, Copenhagen, Denmark; 5 Department of Clinical Biochemistry, Aarhus University Hospital, Aarhus, Denmark; 6 Department of Molecular Medicine, University of Southern Denmark, Denmark

## Abstract

CD163 is the macrophage receptor for uptake of hemoglobin-haptoglobin complexes. The human receptor can be shed from the macrophage surface owing to a cleavage site for the inflammation-inducible TACE/ADAM17 enzyme. Accordingly, plasma ‘soluble CD163’ (sCD163) has become a biomarker for macrophage activity and inflammation. The present study disclosed that 10% of sCD163 in healthy persons is actually extracellular vesicle (EV)-associated CD163 not being cleaved and shed. Endotoxin injection of human volunteers caused a selective increase in the ectodomain CD163, while septic patients exhibited high levels of both soluble ectodomain CD163 and extracellular vesicle (EV) CD163, the latter representing up 60% of total plasma CD163. A poor prognosis of septic patients measured as the sequential organ failure assessment (SOFA) score correlated with the increase in membrane-associated CD163. Our results show that soluble ectodomain CD163 and EV CD163 in plasma are part of separate macrophage response in the context of systemic inflammation. While that soluble ectodomain CD163 is released during the acute systemic inflammatory response, this is not the case for EV CD163 that instead may be released during a later phase of the inflammatory response. A separate measurement of the two forms of CD163 constituting ‘soluble CD163’ in plasma may therefore add to the diagnostic and prognostic value.

CD163 is a transmembrane scavenger receptor expressed exclusively on macrophages and monocytes, and it is pertinent to the clearance of cell-free hemoglobin after intravascular hemolysis^1^,^2^,^3^. CD163 has also been proposed to be involved in regulating tissue regeneration by binding to TWEAK^4^,^5^ and in erythropoiesis by interaction with erythroblasts^6^. Moreover, several reports have identified CD163 as a receptor for bacteria, and as an entry point for viruses^7^,^8^,^9^.

A soluble form of CD163 (sCD163) has been identified in plasma^10^, and it was subsequently unveiled that CD163 may be released from tissue macrophages and monocytes by a metalloprotease-dependent shedding pathway^11^ that is known to involve the inflammation-inducible enzyme TNF-α converting enzyme (TACE/ADAM17)^12^,^13^, pointing towards sCD163 as a potential inflammatory mediator. Accordingly, the plasma concentration of sCD163 has previously been found to be elevated in a number of different inflammatory disorders as reviewed in refs 14,15.

The inflammation-dependent release of sCD163 in plasma has previously been demonstrated in healthy humans, in which intravenous injection of *Escherichia coli* endotoxin caused a rapid increase in the sCD163 concentration^12^. The instant increase in plasma sCD163 was accompanied by a simultaneous increase in TNF-α, which, similarly to CD163, is released from macrophages by TACE/ADAM17-mediated cleavage of its membrane-bound pro-form^16^,^17^. Both proteins peaked after 1.5 h; however, while TNF-α was removed from circulation after 3 h, the endotoxin-induced increase in sCD163 persisted for more than 24 h. As such, sCD163 has been proposed as a surrogate marker of TNF-α□□ and elevated plasma levels have been found to correlate with disease severity in disorders associated with systemic inflammation^1^. To date, the physiological role of sCD163 remains unknown, although some studies have suggested that release of sCD163 blocks the heme iron supply to hemolytic bacteria and trypanosomes by forming a stable complex between sCD163 and the haptoglobin-hemoglobin complex^18^,^19^. Moreover, sCD163 has been proposed to inhibit activated T lymphocyte proliferation^20^ and to promote recognition and phagocytosis of Staphylococcus aureus^21^.

The CD163 protein is expressed in all mammalian species, in which it may play an important role in hemoglobin metabolism, although subtle interspecies differences have been described in regard to ligand preference and regulation of CD163 surface expression. In mice, the CD163-mediated uptake of hemoglobin is effectively mediated independent of haptoglobin-hemoglobin complex formation^22^. Another difference is the mechanism associated with shedding of sCD163 to human and mouse plasma. Both human and mouse sCD163 appears in healthy or naïve plasma respectively, but while human sCD163 is rapidly increased in the circulation upon endotoxin-dependent activation of TACE/ADAM17, mouse sCD163 levels remains stable^13^. As recently published, human CD163 and proTNF-α share a palindromic amino acid sequence (Arg-Ser-(Ser)-Ser-Arg) in their juxta-domain region required for efficient cleavage by TACE/ADAM17 and release of the ectodomain structure^13^. The importance of this sequence was further substantiated by unaltered levels of mouse sCD163 in plasma of ADAM17 deficient mice and by the fact that knock-in of this sequence in mouse CD163 resulted in TACE/ADAM17-dependent shedding comparable to that of human CD163.

In the present study we sought to further explore the molecular nature of plasma CD163, and found that a minor portion of sCD163 in plasma in healthy humans is a membrane-associated form associated with exosomes. This is consistent with previous studies showing that macrophage-derived exosomes constitute a large cohort of circulating extracellular vesicles (EVs) in blood and that mononuclear phagocytes, after platelets, represent the most dominant source of these particles^23^. Exosomes are EVs with a size of 30–150 nm and they are generally believed to originate from inward budding of the membrane in late endosomes in cells forming multivesicular bodies that subsequently are released to the extracellular space by fusion with the plasma membrane. Other EVs include the more heterogeneous microvesicles (50 nm–1500 nm) and apoptotic bodies (50–2000 nm). In contrast to exosomes, microvesicles are formed by budding of vesicles from the plasma membrane independent of intracellular vesicle formation^24^. So far no specific protein markers has been found that allow distinction between the different subtypes of EVs. However it has been shown that exosomes are often enriched in tetraspanins such as CD9, CD63 and CD81^25^. Recent work has shown that macrophages upon either infection with intracellular pathogens or stimulation by endotoxin release not only chemokines and cytokines but also a high amount of exosomes^26^. This prompted us to initiate a deeper characterization of the composition of plasma sCD163 in healthy individuals, in endotoxin-injected individuals, and in septic patients.

## Results

### Characterization of CD163 expression on extracellular vesicles in plasma

**Figure 1:**
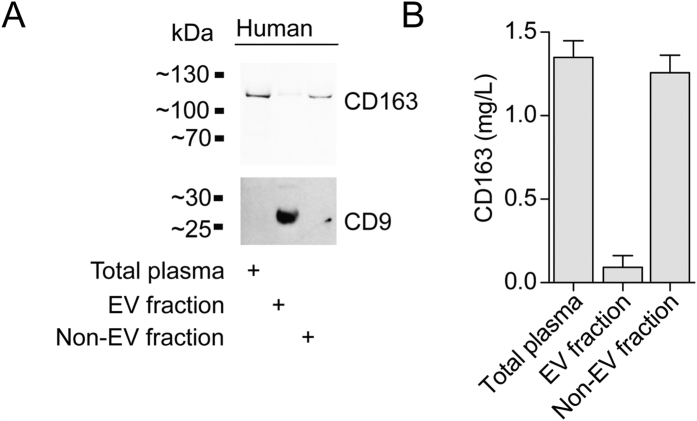
CD163 is expression on EVs in human plasma. (**A**) Western blot analysis of CD9 and CD163. Blots are cropped for presentation. Full-length blots are available in Supplementary Fig. 3. (**B**) CD163 ELISA of human plasma before (total plasma/soluble CD163) and after (Non-EV fraction/soluble ectodomain CD163) precipitation of EVs (EV fraction/EV CD163). Data presented as median with interquartile range.

To investigate if sCD163 in human plasma in addition to the shed ectodomain CD163 also features extracellular vesicle associated CD163, we analyzed CD163 expression in the EV fraction of healthy human plasma (Fig. 1). Western blotting and ELISA analyses against the extracellular domain of CD163 in EVs using a polymer-based precipitation approach (Exoquick) showed that a minor fraction (0.16 g/L, 95% CI: 0.054–0.261 g/L) of total sCD163 (1.35 g/L, 95% CI: 1.13–1.56) in healthy plasma co-precipitates with EVs and CD9 (Fig. 1A,B).

**Figure 2:**
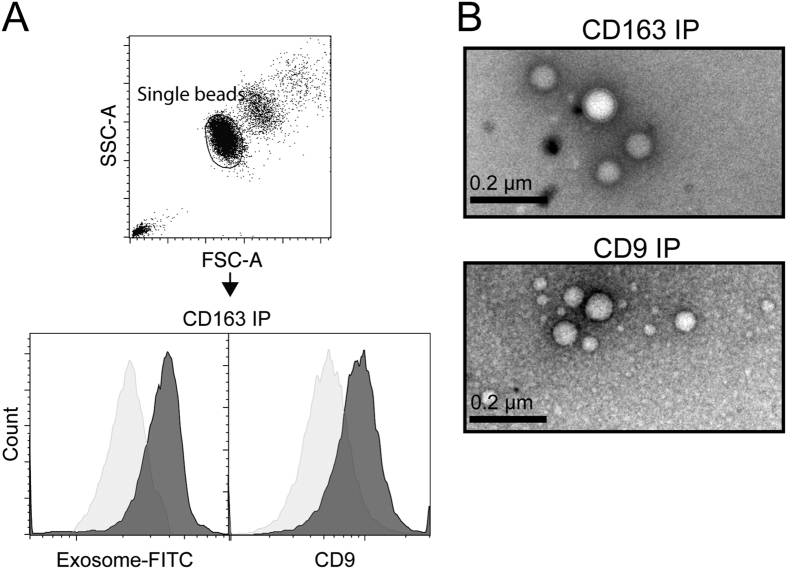
Membrane CD163 is associated with exosomes. (**A**) Beads-based FACS analysis of exosome-FITC and CD9-PE staining on αCD163 (dark grey) or Isotype IgG immuno-precipitated EV’s isolated from human plasma. (**B**) Negative-stain EM of exosomes purified by αCD163 or αCD9 immunoprecipitation of EV’s isolated from human plasma.

To further analyze the association of CD163 with the Exoquick-precipitated plasma fraction (EV fraction), we performed a bead-based FACS analysis of the EV fraction using αCD163 IgG as capture antibody (the same GHI/61 mAb used for detection in the ELISA) and isotype-matched IgG as control (Fig. 2A). Incubation of the EV fraction with αCD163-beads showed specific staining for EVs (Exosome-FITC) and co-staining for CD9. This indicates a co-expression of CD163 and CD9 on the same vesicles and suggests an exosomal origin. Negative staining electron microscopy on αCD163 immuno-precipitated particles confirmed the precipitation of EVs which had an exosome-like appearance with sizes of >100 nm (Fig. 2B).

The presence of CD163 in exosomes implies that the protein remains membrane-bound. This assumption was further verified by the Triton X-114 (Tx-114) method. At temperatures above 30 °C, a Tx-114-containing sample separates into two phases allowing to divide the sample into a detergent (Tx-114) -precipitated membrane protein-rich phase and an aqueous soluble protein-rich phase. As expected, when employing this approach on transfected cells expressing recombinant CD163, the membrane-bound CD163 from cell lysate was recovered in the Tx-114 detergent phase. In contrast, ectodomain sCD163, which is released to the supernatant after PMA induced shedding by ADAM17/TACE, was recovered in the Tx-114 aqueous phase. (Fig. S1).

### Effect of endotoxin injection on ectodomain CD163 and EV-CD163 in plasma

**Figure 3:**
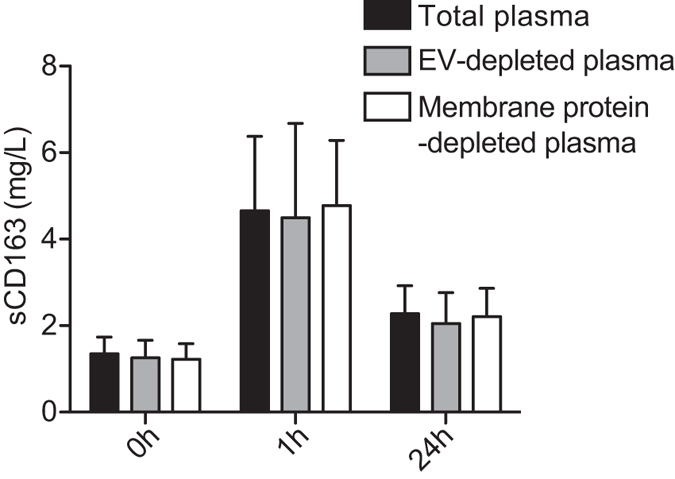
Human endotoxemia induces a release of soluble ectodomain CD163. Levels of sCD163 measured by CD163 ELISA in total plasma, EV depleted plasma (Exoquick treated) and membrane protein depleted plasma (aqueous phase from Tx-114 treatment) in human volunteers (n = 15, males, age 20–27, mean 22.8) after an *i.v.* bolus injection of endotoxin (4 ng/kg). Data presented as median with interquartile range prior to, 1 h and 24 h after intravenous endotoxin injection.

Tx-114 precipitation and Exoquick separation were subsequently applied on human plasma of 15 healthy human volunteers before and after endotoxin injection. As shown in Fig. 3 pretreatment with Exoquick and Tx-114 did not affect the measured level of sCD163 in aqueous supernatant as compared to non-treated plasma. This confirms that the immediate endotoxin-induced increase in sCD163 is a result of shedding of ectodomain CD163 from the surface of macrophages.

### Analysis of ectodomain CD163 and EV-CD163 in plasma from critically ill patients diagnosed with sepsis

**Figure 4:**
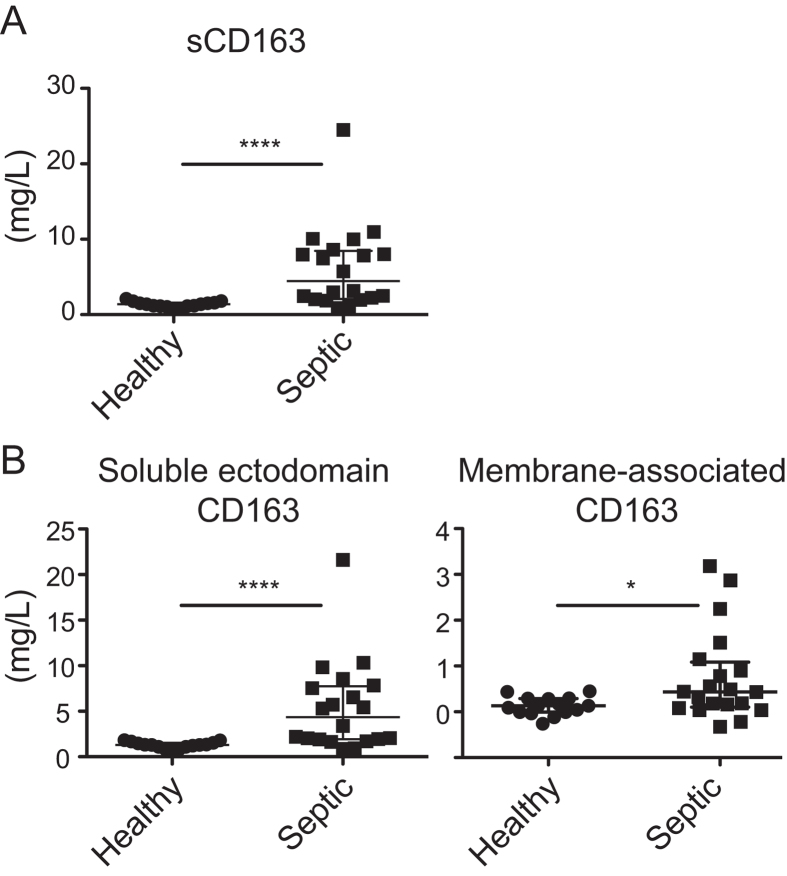
EV-CD163 is increased in patients with sepsis. Total sCD163 (untreated plasma), soluble ectodomain CD163 (aqueous phase of Tx-114-treated plasma) and membrane-associated CD163 (detergent phase of Tx-114-treated plasma) in plasma of the healthy volunteers vs. patients (Table 1) with severe sepsis. Data presented as median with interquartile range and *p < 0.05 and ****p < 0.0001 in non-parametric Mann-Whitney t-test.

**Figure 5:**
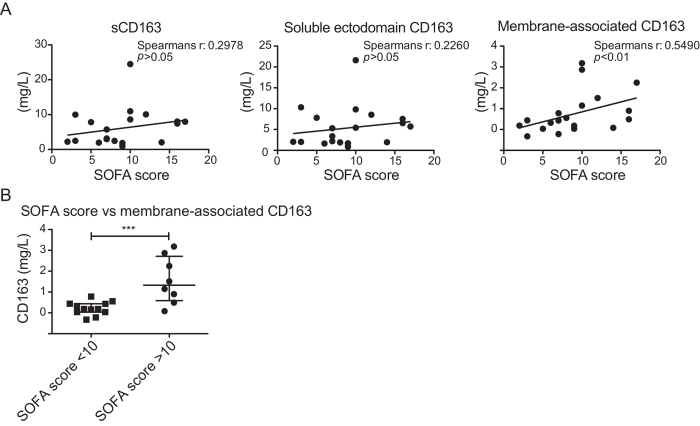
EV-CD163 correlates with SOFA score in septic patients. (**A**) Correlation of total sCD163 (left) soluble ectodomain CD163 (middle) and membrane-associated CD163 (right) level measured by CD163 ELISA in plasma and SOFA score. Correlation was tested by Spearman’s non-parametric correlation analysis. (**B**) membrane-associated CD163 levels in plasma of septic patients with SOFA score <10 (mortality risk <50%) and SOFA ≥ 10 (mortality risk >50%). Data presented as median with interquartile range and ***p < 0.001 as tested by the non-parametric Mann-Whitney t-test.

In addition, we also investigated the plasma of 20 patients with various forms of sepsis (Table 1) as this patient group is among those with the highest reported levels of sCD163 in plasma^1^. Moreover, the increase in sCD163 during sepsis has been suggested to relate directly to the activity of M2 type macrophages^27^. As expected, an elevated level of sCD163 (6.11 mg/L; SD 5.5; Range 0.89–24.48) was seen in the septic patients as compared to healthy individuals (1.35 mg/L; SD 0.39; Range 0.66–2.11) (Fig. 4A). This increase was mainly owing to increase in the sCD163 ectodomain fraction (Fig. 4B left) however, elevated levels of the membrane CD163 fraction was also detected (Fig. 4B right). Moreover, the level of membrane CD163 measured in plasma of septic patients correlated with the level of CD163 associated with EV’s (Fig. S2A). This indicates that the measured increase in membrane CD163 is owing to increased release of exosomes from macrophages containing CD163. A significant increase in membrane CD163 was detected in septic patients with high leukocyte counts >11 × 10^9^/L, (Fig. S2B). Further we observed that the increase in membrane CD163 also correlated with severity of the disease as estimated by the SOFA score (Spearmans r: 0.549, p < 0.01). In contrast, no correlation with sCD163 (Spearmans r: 0.2978, p > 0.05) or ectodomain sCD163 (Spearmans r: 0.226, p > 0.05) (Fig. 5A) was observed. Interestingly, the correlation between SOFA score and membrane CD163 also identified a significant increase in membrane CD163 in septic patients with a mortality rate >50% (SOFA score ≥10) as compared to patients with a mortality rate <50% (SOFA score < 10) (Fig. 5C).

### EV-CD163 in mouse models of experimental endotoxemia, sterile peritonitis and bacteraemia

**Figure 6:**
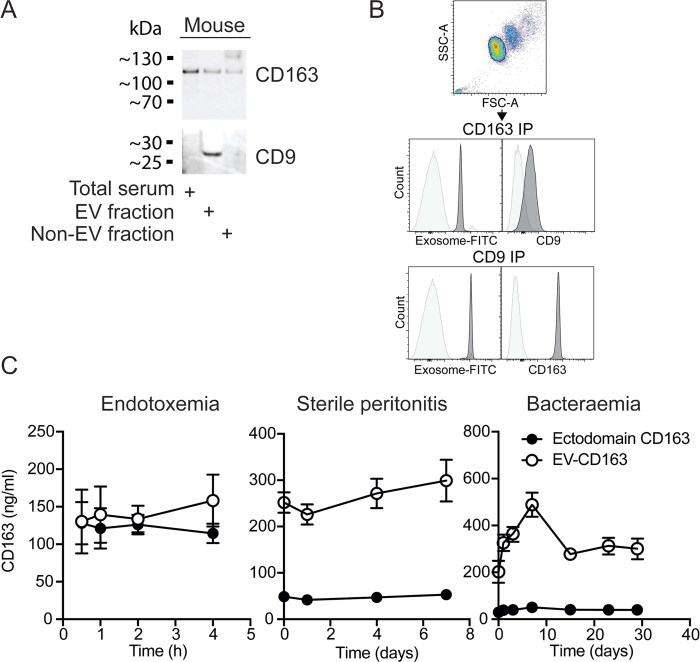
EV-associated CD163 in mice. (**A**) CD9 and CD163 Western blot analysis of mouse plasma before (total serum) and after (non-EV fraction) precipitation of EVs (EV-fraction). Blots are cropped for presentation. Full-length blots are available in Supplementary Fig. 3. (**B**) Beads-based FACS analysis of exosome-FITC and CD9 or CD163 co-staining on αCD163/αCD9 (dark grey) or isotype IgG immuno-precipitated EVs isolated from mouse plasma. (**C**) Levels of ectodomain sCD163 and EV-CD163 measured by mouse CD163 ELISA in Tx-114 phase-separated plasma of mice (n = 6) in models of endotoxemia, sterile thioglycollate-induced peritonitis and *Listeria Monocytogenes* bacteraemia.

To explore if EV-CD163 is also present in mouse plasma we analyzed the expression of CD163 on the CD9 positive EV fraction in plasma of naive mice (Fig. 6). Both western blotting and bead-based FACS analysis confirmed expression of CD163 on CD9-EV’s as previously shown in human plasma (Fig. 6A,B). To further examine the regulation of EV-CD163 in mice we analyzed the plasma levels of ectodomain CD163 and EV-CD163 in mouse models of experimental endotoxemia, sterile peritonitis and bacteraemia. As in humans, the level of EV-CD163 in plasma were not increased in mice subjected to acute inflammation (Fig. 6C left) nor was it increased in mice with sterile peritonitis (Fig. 6C middle). In contrast, when inoculating mice *i.p.* with sublethal levels of *Listeria monocytogenes* increased levels of EV-CD163 was detected after 1day with peak levels 7 days after inoculation (Fig. 6C right).

## Discussion

Since the identification of sCD163 as a natural occurring protein human plasma in the early 2000’s, a large number of studies have reported an association between plasma sCD163 levels and disease severity/progression in various systemic inflammatory conditions^1^. In the present study, we demonstrate that close to 10% of circulating sCD163 exists as a membrane-bound protein associated with EV’s in healthy humans in the absence of systemic inflammation. Using EM and flow cytometry, we found that EV CD163 is associated with exosome-sized particles that co-express the tetraspanin CD9.

The literature on CD163 as a plasma biomarker for macrophage activity is comprehensive and the term ‘soluble CD163’ abbreviated ‘sCD163’ for the CD163 fraction measured in plasma is the general term used despite that we now show that only a major subfraction of sCD163, constituted by the ectodomain CD163, is strictly water-soluble from a biochemically point of view. However, for pragmatic reasons we propose that ‘sCD163’ also forwardly defines the entire plasma CD163 fraction and that ‘soluble ectodomain CD163’ and ‘EV-CD163’ are the terms for the two separate fractions of plasma CD163/sCD163.

Although macrophages have been shown to increase the release and alter the content of exosomes in response to endotoxin *in vitro*^26^, we found that the levels of EV-CD163 remained stable in plasma of human volunteers injected with endotoxin. Instead, a high increase was seen in soluble ectodomain CD163, which is in accordance with an endotoxin-mediated activation of ADAM17/TACE. In patients diagnosed with sepsis we observed a different pattern. Here, the substantial increase in plasma sCD163 was a result of elevated levels of both soluble ectodomain CD163 and EV-CD163. The significant difference between the groups was not affected by correcting for the minor influences by gender and age^14^,^28^. Correlational analyses furthermore revealed a significant correlation between exosome CD163 with SOFA score, while no such correlation was found between either total sCD163 or ectodomain CD163 and SOFA score. Furthermore, the highest levels of exosome CD163 was measured in patients with a SOFA score ≥10.

In mice lacking the motif for TACE/ADAM17-mediated shedding of CD163^13^, exosome CD163 made up more than 50% of total plasma CD163. However, the overall level of plasma CD163, is much lower in mice compared to humans, although the absolute amount of exosome-bound CD163 is at the same level in both species. In accordance with the human data, we observed that mice with induced sepsis by injection of a non-lethal dose of *Listeria monocytogenes* experienced a high and long-term increase in exosome CD163 with peak values 7 days after infection. In contrast, no increase in exosome CD163 was detected in experimental endotoxemia and non-infectious sterile peritonitis.

The experimental setup in the present study does not allow us to draw any conclusions regarding the function of exosome CD163. However, a mounting volume of evidence suggests that myeloid cell-derived exosomes are important in cell-to-cell communication between antigen-presenting cells, thus further stressing the putative involvement of exosome CD163 in mediating inflammatory processes. For instance, microRNA (miRNA) has been shown to be transported from host to recipient cells by exosome-mediated delivery^29^. miRNA plays an important role in modulating the cellular transcriptome and exosomal delivery, e.g. of miRNA-33 that is known to orchestrate macrophage metabolism and thus polarization^30^. This stimulation could permit macrophages to stimulate recipient cells to achieve a more pro-inflammatory phenotype. In dendritic cells, exosome miRNA-155 alters the response of bone marrow derived dendritic cells to endotoxin^31^. Uptake of exosome/microvesicle CD163 can also explain a previous study reporting that sCD163 in complex with IgG can be taken up via the FcgR receptor in monocytes, which then recycles the endocytosed CD163 to the cell surface^32^. As CD163 is often described as being associated with an anti-inflammatory macrophage phenotype it can be speculated that the release of CD163+ exosomes is part of an anti-inflammatory response aiming at repolarizing activated macrophages to the CD163-positive anti-inflammatory macrophages.

In conclusion, the present study shows that soluble ectodomain CD163 and EV-CD163 are components of separate macrophages responses in the context of systemic inflammation. Injection of endotoxin in healthy human human subjects, which models an acute systemic inflammatory response similar to that of the very early stages of sespsis^33^, only activates release of the ectodomain CD163. In contrast, increased levels of exosome CD163 was detected in the septic patients thus suggesting a release of EV-CD163 during a later and more advanced phase of the inflammatory response. In future patient studies, where sCD163 in plasma is included, it may therefore add to the prognostic value to separate the sCD163 into its two plasma subcomponents, soluble ectodomain CD163 and EV-CD163.

## Methods

### Participants

The human experiments were approved by the Research Ethical Committee of Copenhagen and Frederiksberg Municipalities, Denmark (protocols no. H-A-2009–020, H-2–2009–131, and H-1-2010-20), and were conducted in accordance with the Declaration of Helsinki and informed consent was obtained from all subjects. Intravenous injection of human volunteers with endotoxin (4 ng/kg) was carried out as previously reported^34^, and data are reported for baseline, and 1 h and 24 h post-injection in the present study. Samples from adult patients with diagnosed sepsis admitted to intensive care where obtained from previously published studies on cerebral hemodynamics and the systemic inflammatory response^33^,^35^,^36^,^37^. Enrollment of patients was limited by exclusion criteria affecting hemodymamics such as evidence of neurotrauma, cerebrovascular disease, neuroinfection, or pregnancy and arterial hypertension. The onset of the disease and disease focus were not included as enrollment criteria. SOFA score was calculated on admission to the intensive care unit in accordance with standardized guidelines^38^,^39^.

### EV preparation

EVs were purified from human or mouse plasma using the Exoquick Exosome Precipitation solution in accordance with manufactures instruction (Systems Biosciences, Palo Alto, CA, USA). In brief, plasma was centrifuged at 3000 *g* for 15 min at 4 °C to remove debris and exoquick was mixed in plasma:exoquick ratio 4:1 by gentle inversion of the tube. Exoquick/plasma mix was subsequently centrifuged at 3000 *g* for 15 min at 4 °C after which supernatant was recovered and pelleted EVs were resuspended in appropriate buffer for downstream applications.

### Triton X-114 assay

Membrane-associated and ectodomain CD163 were separated using the Triton X-114 method (Tx-114, Sigma-Aldrich, Brøndby, Denmark)^40^. In short, plasma and cell lysate were precleared to remove cellular debris using differential centrifugation step (30 min at 2000 g followed by 45 min centrifugation of resulting supernatant at 12000 g). Subsequently samples were diluted 1:10 in a precondensed 2% Tx-114 solution in 1x PBS pH 7.4 and incubated for 10 min on ice. Samples were then heated to 37 °C for 10 min in order to induce separation of the aqueous and the detergent phase after which samples were centrifuged at 22,500 g for 10 min at 37 °C. The aqueous phase were then harvest and kept at 4 °C for later analysis. The detergent phase were further washed twice with 0.1% Tx114 after which protein was harvested for analysis by chloroform:methanol (ratio 4:1) precipitation. Precipitated protein was subsequently resuspended in 1x LDS sample buffer (Thermo Fischer Scientific, Slangerup, Denmark) by boiling.

### ELISA and Western blotting

Levels of human and mouse CD163 were quantified by ELISA as previously described^22^,^41^. For western blotting exoquick precipitated EVs were suspended in 1xRIPA buffer complemented with cOmplete Mini, EDTA-free protease inhibitor cocktail (Roche Applied Science, Hvidovre, Denmark) and samples were separated by SDS-PAGE using a 12% NuPAGE Bis-Tris gel and MES running buffer (Invitrogen) followed by blotting on a PVDF membrane using the iBlot dry blotting system (Thermo Fischer Scientific). Immunoblotting was performed using specific antibodies for CD9 and CD163 (pAb rabbit anti-human CD9; Systems Bioscience, mAb rat anti-mouse CD9 (clone MF1); Bio-Rad, UK, mAb mouse anti-human CD163 (clone Mac2–158); IQ products, Netherlands and pAb rabbit anti-mouse CD163)^22^ and a secondary HRP conjugated goat anti-rabbit, rat or mouse IgG antibody (Sigma-Aldrich). Immunoreactive bands were visualized on a FUJI FLA3000 gel doc (Fujifilm Europe GmBH, Düsseldorf, Germany) using an ECL substrate (Pierce, Rockford, IL, USA) and a 240 sec exposure time.

### Flow cytometry and electron microscopy

Flow cytometric analysis of Exoquick precipiated EVs was done using the Exo-FLOW exosome purification kit (Systems Biosciences) in accordance with the manufactures instruction. In short, Exo-Flow streptavidin magnetic beads were incubated with biotinylated antibody against human CD163 (clone GHI/61; isotype mouse IgG2A, Biolegend, London, UK), mouse CD163 (Clone 3E10B10; rat IgG2A)^22^ or isotype control (Biolegend) for 1 h, washed and incubated with exoquick precipitated exosomes O/N at 4 °C in end-over end mixer. Beads were washed and resuspended in FACS buffer (1x PBS pH 7.4, 1 mM EDTA, 3% FCS) supplemented with Fc Receptor blocking antibody and stained for exosomes using Exo-FITC (Systems Biosciences) or CD9-PE (anti-human CD9; Clone HI9a, dilution 1:400, or anti-mouse CD9; Clone MZ3, dilution 1:400, Biolegend) and analyzed on a BD LSR II flow cytometer (BD Bioscience, France). Exo-Flow streptavidin magnetic beads were gated in Forward (FSC) vs. Side scatter (SSC) and analyzed for FITC and PE fluorescence in histogram. All analysis were done in FlowJo X (v10.0.8). Subsequently anti-CD9 and anti-CD163 precipitated EVs were subjected to electron microscopic analysis by eluting EVs from Exo-Flow streptavidin magnetic beads by incubation in Exosome Elution buffer (Systems Bioscience) for 2 h at RT. Electron microscopic analysis was performed using the negative staining method. Briefly, 3 μL drops of eluted EVs were adsorbed onto Formvar/carbon-coated copper grids for 10 min. Grids were then washed with water and negatively stained with 2.0% phosphotungstic acid in distilled water for 30 sec. Grids were examined with a transmission electron microscope at 200 kV (Tecnai G2 FEI, Netherlands). Images were taken with a digital camera (Veleta, Olympus, Japan) using the software TIA (Tem Imaging Analysis).

### Mouse models

8 weeks old C57BL/6 J mice were purchased from Taconic (Ry, Denmark) or Janvier Labs (Le Genest-Saint-Isle, France) and housed under 12 h/12h day-night cycle with ad libitum water and food. **Endotoxemia**: Mice were injected i.v. with 2.5 mg/kg endotoxin (endotoxemia) and blood were collected after 0.5 h, 1 h, 2 h and 4 h. Data presented are a reanalysis of previously published samples^13^. **Sterile peritonitis**: Mice were injected *i.p.* with 1 ml 3% thioglycollate as described in^42^,^43^ and blood was collected prior and 1–7 days after injection. **Bacteraemia**: Mice were *i.p.* inoculated with 1 × 10^4^ CFU of *Listeria Monocytogenes* as described by^44^^44^ and blood was collected prior and 7–35 days after inoculation. All animal experiments were approved and carried out in accordance with the limiting principles for using animal in testing (the three R’s, replacement, reduction and refinement) and approved by either the Danish Animal Welfare Committee or the ethical committee of the French Ministry of Higher Education and Research.

### Statistical analysis

All statistical analyses were performed in GraphPad Prism 5.0f for Mac OS X. Differences between groups were analyzed for statistical significance using the Mann-Whitney U-test and correlation analysis was performed using Spearmans non-parametric correlation test. Data was considered statistical significant at p < 0.05.

## Additional Information

**How to cite this article:** Etzerodt, A. *et al*. Soluble ectodomain CD163 and extracellular vesicle-associated CD163 are two differently regulated forms of ‘soluble CD163’ in plasma. *Sci. Rep.* **7**, 40286; doi: 10.1038/srep40286 (2017).

**Publisher's note:** Springer Nature remains neutral with regard to jurisdictional claims in published maps and institutional affiliations.

## Supplementary Material

Supplementary Dataset 1

## Figures and Tables

**Table 1:** Characteristics and clinical parameters of healthy human volunteers and septic patients.

Characteristics	Values
Healthy volunteers	
No. of patients	15
Age, mean(SD) [Range]	23 (2) [20–27]
*Sex*	
Women	0
Men	15
Septic patients	
No. of patients	20
Age, mean(SD) [Range]	58 (13) [25–75]
*Sex*	
Women	4
Men	16
*Focus (No. Of patients*)	
Lung	6
Blood	3
CNS	5
Abdomen	1
Soft tissue	4
Unknown	1
*Clinical parameters, mean(SD*) [*Range*]	
APACHE II score	23.6 (8.107) [5–36]
SAPS score	49.85 (13.99) [21–78]
SOFA score	9 (4.341) [2–17]
Temperature (°C)	37.4 (0.98) [36.2–40.1]
Leukocyte count (10^9^/L)	15.04 (15.49) [1–49.4]
CRP (mg/L)	124.1 (99–36) [0–330]
