# Supplementary material for: Soluble ectodomain CD163 and extracellular vesicle-associated CD163 are two differently regulated forms of ‘soluble CD163’ in plasma
Source: Sci Rep. 2017 Jan 13;7:40286. doi: 10.1038/srep40286 (PMC5234032; doi:10.1038/srep40286)
Supplement: Supplementary Dataset 1 [file srep40286-s1.doc]

**Supplementary figures**

**Soluble ectodomain CD163 and extracellular vesicle-associated CD163 are two differently regulated forms of ‘soluble CD163’ in plasma**

Anders Etzerodt^1,2^, Ronan M. G. Berg^3^, Ronni R. Plovsing^4^, Morten N. Andersen^1,5^, Magali Bebien^2^, Mohamed Habbeddine^2^, Toby Lawrence^2^, Holger J. Møller^5^, Søren K. Moestrup^1,5,6,*^

^1^Department of Biomedicine, University of Aarhus, Aarhus, Denmark

^2^Centre d’Immunologie de Marseille-Luminy, Aix Marseille Université UM2, Inserm, U1104, CNRS UMR7280, Marseille, France

^3^Centre of Inflammation and Metabolism, Rigshospitalet, Copenhagen, Denmark

^4^Department of Intensive Care, Rigshospitalet, Copenhagen, Denmark

^5^Department of Clinical Biochemistry, Aarhus University Hospital, Aarhus, Denmark

^6^Institute of Molecular Medicine, University of Southern Denmark, Denmark

**Figure S1. Precipitation of membrane-associated CD163 by the Triton X-114 phase separation method.** HEK293 cells stably expressing human CD163 were stimulated with PBS or PBS+PMA for 1h to induce TACE/ADAM17 mediated shedding. Cell lysate and tissue culture supernatant were analyzed by western blotting for CD163 with (right) or without (left) Trition X-114-mediated phase separation into an aqueous phase (soluble proteins) and detergent phase (membrane anchored proteins).

**Figure S2. Membrane-associated CD163 correlates with level of EV-CD163 and is elevated in septic patients with a high total white blood cell count.** (**A**) Correlation between plasma level of membrane associated CD163 (Tx-114 detergent phase) and EV-associated CD163 (EV precipitated plasma) as measured by CD163 ELISA. Correlation was tested by Spearman’s non-parametric correlation analysis. (**B**) Membrane-associated and soluble ectodomain CD163 levels in plasma of septic patients with leukocyte count <11 x10^9^ cells/L (Leuko<11) and leukocyte count ≥11 x10^9^ cells/L (Leuko≥11). Data presented as median with interquartile range and * p<0.05 as tested by the non-parametric Mann-Whitney t-test.


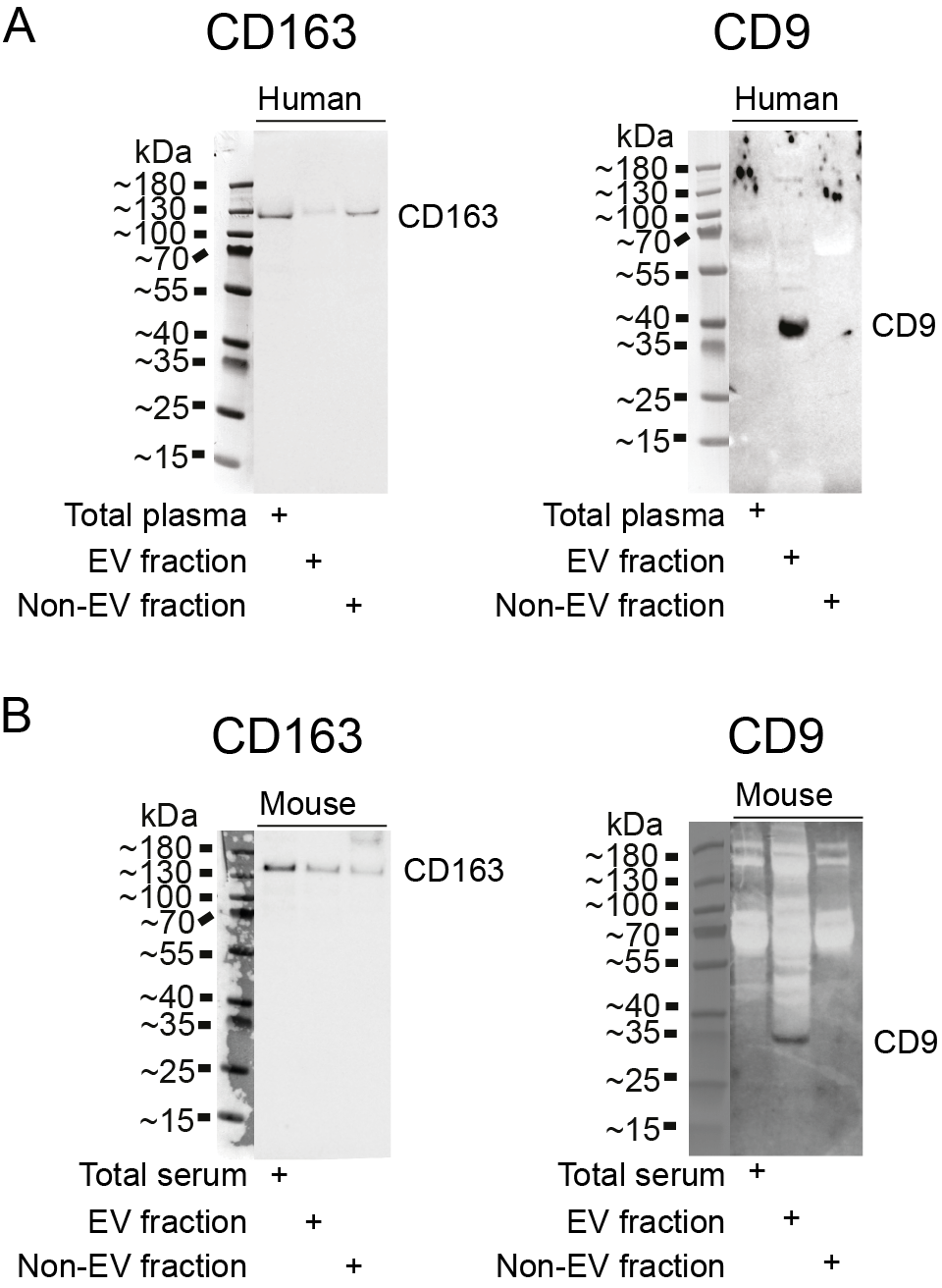


**Figure S3. Full length blots of human and mouse CD163 and CD9.** CD9 and CD163 Western blot analysis of human plasma (**A**) and mouse (**B**) serum before (total plasma/serum) and after (non-EV fraction) precipitation of EVs (EV-fraction).
